# Acupuncture Combined with Three-Step Analgesic Drug Therapy for Treatment of Cancer Pain: A Systematic Review and Meta-Analysis of Randomised Clinical Trials

**DOI:** 10.1155/2021/5558590

**Published:** 2021-07-26

**Authors:** De-hui Li, Yi-fan Su, Huan-fang Fan, Na Guo, Chun-xia Sun

**Affiliations:** ^1^Hebei Province Hospital of Chinese Medicine, The First Affiliated Hospital of Hebei University of Chinese Medicine, Shijiazhuang 050011, China; ^2^Graduate School of Hebei University of Chinese Medicine, Shijiazhuang 050091, China

## Abstract

**Objective:**

The purpose of this study was to systematically evaluate the efficacy and safety of acupuncture combined with the WHO three-step analgesic drug ladder for cancer pain.

**Methods:**

The Cochrane Library, PubMed, and CNKI Database of Systematic Reviews were searched. Using the Cochrane Register for Randomized Controlled Trials, the quality of the included literature was evaluated, and the meta-analysis was carried out with RevMan 5.3 software.

**Results:**

Compared with three-step analgesia alone, acupuncture combined with three-step analgesia for cancer pain increased pain relief response rates (RR = 1.12, 95% CI: 1.08∼1.17, *P* < 0.00001), reduced NRS score (SMD = −1.10, 95% CI: −1.86∼−0.35, *P*=0.004), reduced the rate of side effects (RR = 0.45, 95% CI: 0.38∼0.53, *P* < 0.00001), including nausea (*P* < 0.00001), vomiting (*P*=0.008), constipation (*P* < 0.00001), and dizziness (*P*=0.010), reduced the burst pain rate (SMD = −1.38; 95% CI: −2.44∼−0.32, *P*=0.01), shortened analgesia effect onset time (*P*=0.004), and extended the duration of response (*P* < 0.0001).

**Conclusion:**

For the treatment of cancer pain, acupuncture combined with three-step analgesic drugs is better than using only three-step analgesic drugs.

## 1. Introduction

Pain is one of the most debilitating symptoms experienced by patients with advanced cancer. According to WHO statistics, 70% of cancer patients worldwide have some degree of pain in the advanced stages of cancer [1]. Because the pain is intense and easily aggravated, it directly affects the appetite, sleep, psychological status, and treatment effect of patients, reduces their quality of life, and increases their psychological stress [2, 3]. Cancer pain has become a medical, psychological, and social issue of great concern. At present, the treatment of cancer pain mostly utilizes the three-step “ladder” treatment principle proposed by the WHO, where mild, moderate, and severe pain are treated with nonsteroidal anti-inflammatory drugs (NSAIDs), weak opioids, and strong opioids, respectively [4]. Although the analgesic effect of three-step drugs is good, their side effects, such as liver and kidney function damage, risk of dependency and addiction, respiratory inhibition, and gastrointestinal side effects, limit their clinical application [5]. One primary reason why cancer pain is difficult to control is because cancer pain patients cannot tolerate the side effects of analgesics. Therefore, there is a consensus to seek other effective and safe analgesic methods [6]. Acupuncture is an important part of TCM. Acupuncture is to insert a needle at one of the patient's acupoints and use a specific manipulation to stimulate the patient's acupoints to achieve an effect (DE QI). Acupuncture has outstanding performance in the treatment of all kinds of pain through its principle of moving qi, dredging qi channels and collaterals, and activating blood. Various acupuncture treatments can be combined, with no risk of addiction, no side effects, convenient application and at a low cost, demonstrating the unique advantages of TCM in the treatment of cancer pain [7]. In the last 20 years, there have been many clinical reports on the utility and safety of acupuncture for the treatment of cancer pain, and acupuncture therapy is a widely recognised alternative measure for the treatment of cancer pain. Therefore, it is necessary to use a systematic evaluation method to rigorously evaluate the randomised controlled study of acupuncture combined with three-step analgesic drugs to treat cancer pain, to assess its exact effect in the treatment of cancer pain.

## 2. Methods

### 2.1. Data Sources

The following databases were searched from their inception to January 10, 2021: the Cochrane Library, PubMed, Embase, CNKI, China Biology Medicine disc (CBMdisc), Chinese Journal of Science and Technology database (VIP), and Wanfang database. We searched MeSH (Medical Subject Headings) term trees for “acupuncture” and “pain” in PubMed, and the keywords searched included “acupuncture”, “needling”, “tumour”, “cancer”, “neoplasm”, “ache”, “pain”, and “randomised controlled trial”. The keywords were translated into Chinese and searched in the above-mentioned Chinese databases. Search terms were combined with the Boolean “AND” and “OR” terms in search strategies, for example, (“acupuncture” OR “needling”) AND (“cancer” OR “tumour” OR “neoplasm” OR “ache” OR “pain”) AND (“randomised controlled trial”). Comprehensive retrieval was carried out according to the characteristics of different databases. Then, the literature mentioning “randomised controlled” and “randomised grouped” was screened. In addition, we manually searched our own personal literature files. After reading the full text of the included literature and related articles, we collected the documents together in hard copy format for preservation.

### 2.2. Inclusion Criteria

#### 2.2.1. Types of Studies

The included studies were all randomised controlled clinical trials. The published experiments included were mainly in the form of theses and abstracts. There were no restrictions on the language of publication.

#### 2.2.2. Types of Participants

The subjects were patients with malignant tumours confirmed by cytology or histopathology, and all patients had cancer pain. There were no limits on age, gender, race, and nationality of the patients; however, patients had to be able to clearly describe their pain to medical staff.

#### 2.2.3. Types of Interventions

In the literature, the intervention treatment group was treated with acupuncture augmented by three-step analgesia, including traditional acupuncture or other acupuncture methods, such as ear acupuncture and electroacupuncture. Acupuncture points included traditional acupuncture points and pain points. The control groups only received three-step analgesic treatment.

#### 2.2.4. Types of Outcome Evaluations

The included materials had a clear evaluation standard for curative effects and at least one clinical index related to cancer pain, including the effective rate of pain relief after treatment, quality of life score, side effect rates, burst pain rate, onset time to analgesic effect, and duration of response.

### 2.3. Exclusion Criteria

The exclusion criteria were as follows: if patients had one or more other type(s) of pain in addition to cancer pain; if the study used moxibustion, percutaneous electrical stimulation of nerves, acupoint injection, laser irradiation, cupping, massage, herbal medicines, or other intervention measures; if the experiments were carried out on patients during or a few days after surgical therapy, radiotherapy, chemotherapy, or hyperthermia-therapy on their malignant tumours; if the trial design was not rigorous; if inappropriate statistical methods were used; if the paper was only an abstract, review, or summary of previously published literature; if the study has no result indicators; if the experimental design was unreasonable; or if the literature could not be obtained by contacting the author.

### 2.4. Data Extraction and Bias Risk Assessment

Two researchers independently evaluated the quality of each study meeting the inclusion criteria and extracted the data, including the baseline situation, intervention measures, and efficacy results, and cross-checked the data. Any disagreements were resolved through discussion or assessment by a third researcher. We used a “Modification of Cochrane Tool to assess the risk of bias in randomised trials,” where a decision regarding bias must be made, categorised into “probably no” or “probably yes,” for items that are thought to be of unclear risk [8]. We judged trials with more than 2 and more than 4 high-risk components as moderate risk and high risk, respectively [9]. The following criteria were used to assess the risk of bias: whether the study was randomised; how allocation concealment was conducted; whether the study was double-blind or triple-blind; whether the results data were complete; and whether there was selective reporting or other types of bias. The authors categorised studies into “low risk,” “unclear risk,” and “high risk” categories. For dropout patients, we contacted the authors of the studies twice over four weeks via e-mail for missing or unclear data. If missing data could not be found, they were recorded as high risk; if no response was received, the data were marked as unclear risk. All authors reached a consensus on the results of bias risk assessment.

### 2.5. Data Synthesis

The effect of acupuncture combined with three-step analgesic drug therapy for treatment of cancer pain was analyzed in terms of response rate, numerical rating scale (NRS), side effect rates, times of burst pain, onset time, and duration of response (DOR). If the information included in the study was insufficient, we communicated with the main author to obtain accurate data. RevMan 5.3 software provided by the Cochrane Collaboration Network was used for the meta-analysis. The relative risk (RR) was used for the enumeration data, the mean difference (MD) was used for the measurement data, and the 95% confidence interval (CI) was used for each effect quantity. When the heterogeneity of test results was not statistically significant (*P* > 0.05), a fixed effects model was selected; when the heterogeneity of test results was statistically significant (*P* < 0.05), a random effects model was selected. A funnel plot was used to analyse and detect publication bias.

## 3. Results

### 3.1. Study Description

**Figure 1:**
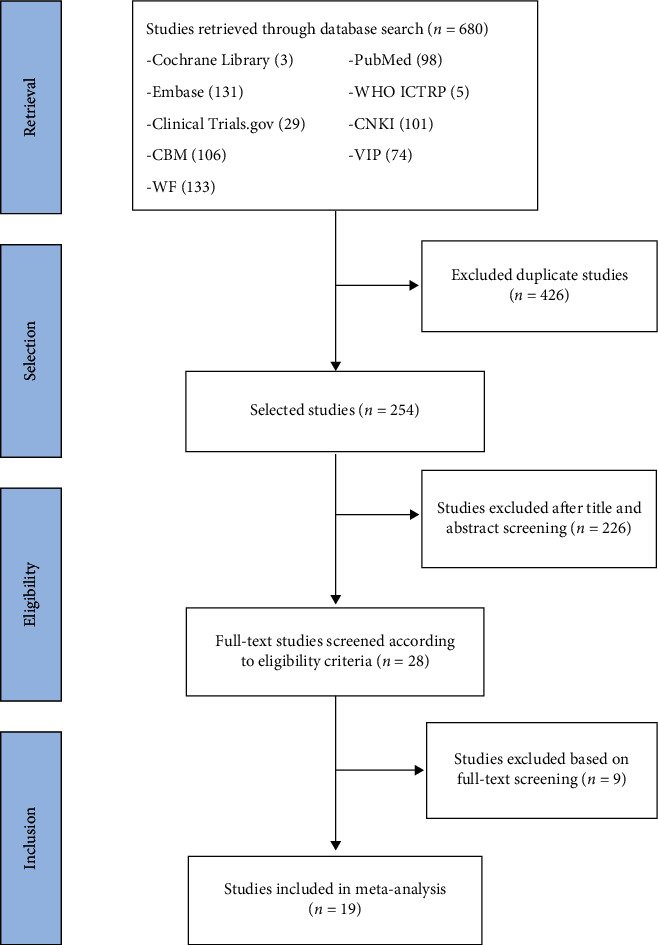
Flowchart of the literature review and selection process.

The first search found 115 potentially relevant articles. After reading and screening, 19 articles met our inclusion criteria (Figure 1). The critical data from all the included RCTs are shown in Table 1 [10–28]. In total, 1502 cancer pain cases were included. The numbers of cases of acupuncture combined with three-step analgesic drug therapy (treatment group) and three-step analgesic drug therapy (control group) were 751 and 751, respectively. All patients' cancers were confirmed by cell histology or pathology, and pain was their main symptom. The baseline was comparable between the two groups. Almost all of the research was on the use of manual acupuncture (AT), which is guided by the theory of TCM for acupuncture interventions. Two studies used electroacupuncture (EA) [11, 28]. One study used floating acupuncture (FA) [12]. Two studies used fire needle (FN) [13, 14]. Three studies used wrist-ankle acupuncture (WA) [17–19]. Among them, the two acupuncture methods were all included in Fu Yang et al.'s report [17], in which morphine hydrochloride sustained-release tablets and acupuncture or wrist-ankle acupuncture were used in the treatment of cancer pain. All studies provided patients with a semistandardised acupuncture programme, that is, the use of a predefined set of acupoints combined with a set of acupoints according to the location of the tumour. The Ashi point, Zusanli (ST36), Hegu (LI4), Sanyinjiao (SP6), and Taichong (LR3) points were most frequently used. For most studies, patients received acupuncture treatment for 1 to 3 weeks, for durations of 20 to 60 min per session. The evaluation criteria for the curative effect were similar across studies. The objective outcome measures were treatment response rate, NRS, side effect rates (nausea, vomiting, constipation, hiccups, dizziness, itching, palpitation, and abdominal distention), times of burst pain, onset time to analgesic effect (min), DOR (h), quality of life (QOL), Karnofsky performance status (KPS), and quality of life questionnaires (QLQ-C30). The minimal important difference (MID) refers to the change in the score of the smallest efficacy evaluation questionnaire recognised by the patient. MID indicates an important improvement in symptoms and signs; the intervention has achieved the minimal important difference.

### 3.2. Risk of Bias

**Figure 2:**
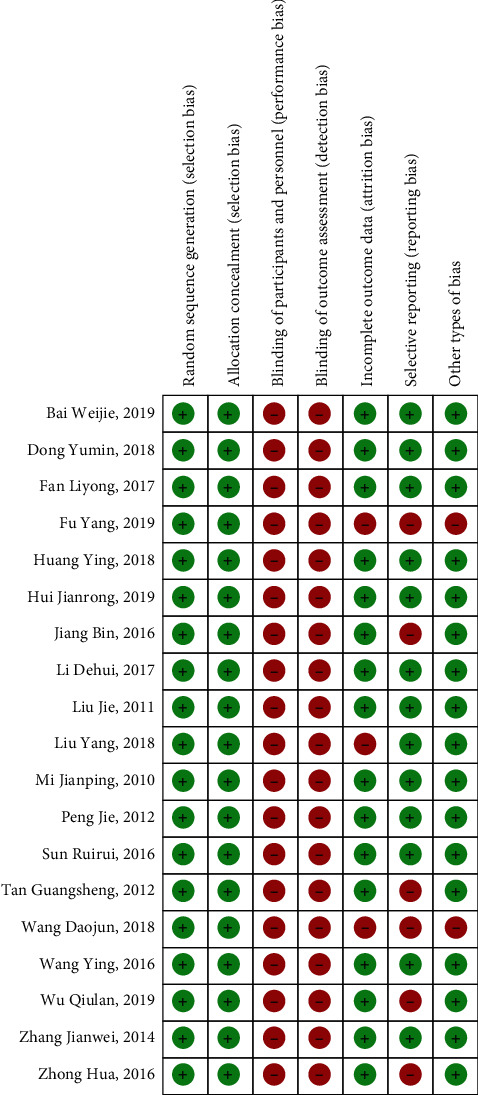
Cochrane risk of bias by trial.

Most included RCTs had a high risk of bias. Nineteen RCTs [10–28] described their randomisation methods. Among them, 9 RCTs [10, 12–14, 16–18, 22, 24] used a random number table, 1 study [15] used a computer-generated random number sequence for randomisation, and 3 RCTs [20, 21, 26] randomly numbered cases according to the order of hospitalization. Three RCTs [10, 15, 17] described incomplete outcome methods, and these three studies had cases of dropouts. Two studies reported details about allocation concealment [15, 16]. Fourteen RCTs described adverse events from acupuncture combined with three-step analgesic drugs [10, 11, 13–23, 26].  Table 2 presents the Cochrane risk of bias assessment of the included articles. There were 2 trials with high risks of bias [10, 17], 5 trials with moderate risk of bias [12, 15, 18, 23, 25], and 12 trials with low risk of bias [11, 13, 14, 16, 19–22, 24, 26–28]. A high risk of bias resulted from lack of blinding of participants and personnel and lack of blinding among outcome assessors. A moderate risk resulted from selective reporting bias and incomplete outcome data, and a low risk of bias resulted from randomisation sequence generation and allocation concealment (see Figure 2).

### 3.3. Response Rates

**Figure 3:**
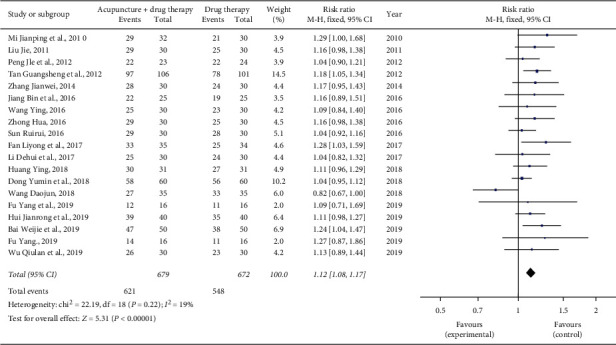
Forest plot of the total response rates of acupuncture combined with three-step analgesic drugs versus three-step analgesic drugs alone for cancer pain.

Eighteen studies reported the response rates to pain relief after treatment [10–14, 16–28]. In the treatment group, 621 out of the 679 cases had effective responses; in the control group, among the 672 cases, 548 had effective responses. The heterogeneity test in the meta-analysis showed that *χ*^2^ = 22.19, *P*=0.22, *I*^2^ = 19%, and there was no significant difference between the studies, so a fixed effects model was used. The total response rate of the treatment group was better than that of the control group, and the difference was statistically significant (*n* = 1351, RR = 1.12; 95% CI: 1.08∼1.17, *P* < 0.00001; see Figure 3).

### 3.4. NRS Score

**Figure 4:**
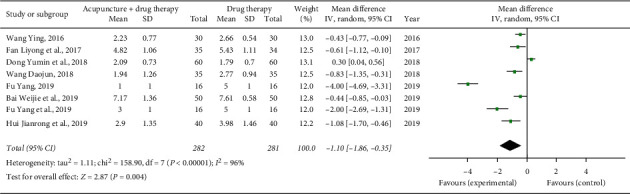
Forest plot of NRS score of acupuncture combined with three-step analgesic drugs versus three-step analgesic drugs alone for cancer pain.

Seven studies reported NRS scores after treatment [10, 11, 14, 17, 19, 22, 24]. Overall, 282 cases were in the treatment group, and 281 were in the control group. The heterogeneity test of the meta-analysis showed that *χ*^2^ = 158.90, *P* < 0.00001, *I*^2^ = 96%, and the differences between the studies were statistically significant, so a random effects model was used. The NRS score of the treatment group was lower than that of the control group, and the difference was statistically significant (*n* = 563, SMD = −1.10, 95% CI: −1.86∼−0.35, *Z* = 2.87, *P*=0.004; see Figure 4).

### 3.5. Side Effect Rates

**Figure 5:**
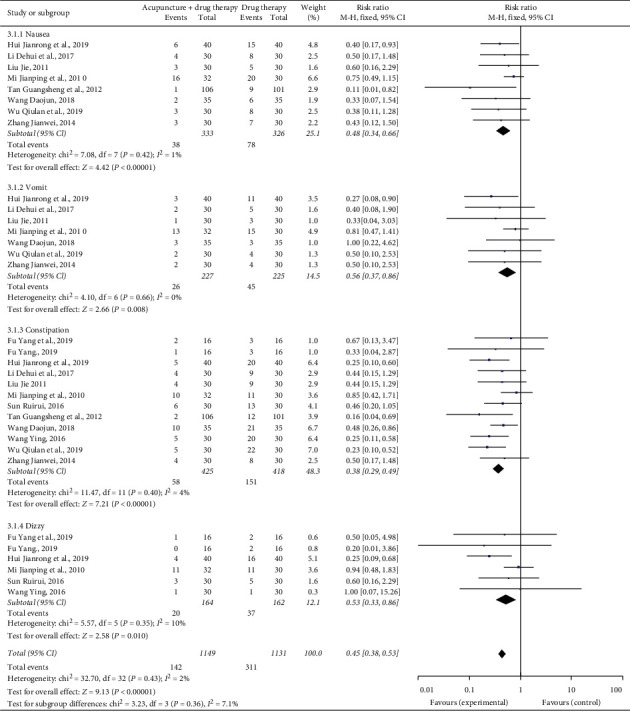
Forest plot of side effect rates of acupuncture combined with three-step analgesic drugs versus three-step analgesic drugs alone for cancer pain.

Side effects mainly included nausea, vomiting, constipation, and dizziness. Eight studies reported the number of cases of nausea [10, 13, 16, 18, 21–23, 26], 7 studies reported the number of cases of vomiting [10, 13, 16, 18, 21, 22, 26], 11 studies reported the number of cases of constipation [10, 11, 13, 16–18, 20–23, 26], and 5 studies reported the number of cases of dizziness [11, 13, 17, 20, 22]. The consolidated statistics results demonstrated that, compared to the control group, in the treatment group, the incidence of nausea (*n* = 659, RR = 0.48, 95% CI: 0.34∼0.66, *P* < 0.00001), vomiting (*n* = 452, RR = 0.56, 95% CI: 0.37∼0.86, *P*=0.008), constipation (*n* = 843, RR = 0.38, 95% CI: 0.29∼0.49, *P* < 0.00001), and dizziness (*n* = 326, RR = 0.53, 95% CI: 0.33∼0.86, *P*=0.010) decreased (see Figure 5).

### 3.6. Burst Pain

**Figure 6:**
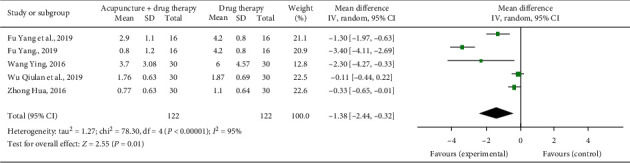
Forest plot of times of burst pain of acupuncture combined with three-step analgesic drugs versus three-step analgesic drugs alone for cancer pain.

Four studies reported the mean number of burst pain events [11, 12, 17, 18]. The heterogeneity test in the meta-analysis showed that *χ*^2^ = 78.30, *P* < 0.00001, *I*^2^ = 95%, and the differences between the studies were statistically significant, so a random effects model was used. The combined statistical results showed that the incidence of burst pain in the treatment group was lower than that in the control group (*n* = 244, SMD = −1.38, 95% CI: −2.44∼−0.32, *P*=0.01; see Figure 6).

### 3.7. Onset Time to Analgesic Effect and Duration of Response

**Figure 7:**
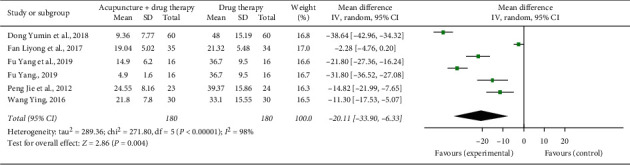
Forest plot of the onset time of acupuncture combined with three-step analgesic drugs versus three-step analgesic drugs alone for cancer pain.

**Figure 8:**
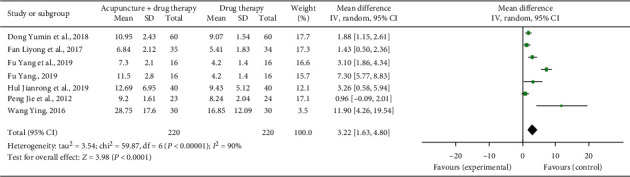
Forest plot of the duration of response of acupuncture combined with three-step analgesic drugs versus three-step analgesic drugs alone for cancer pain.

Five studies reported the mean onset time [11, 17, 19, 24, 28]. The combined statistical results showed that the onset time in the treatment group was shorter than that in the control group (*n* = 360, SMD = −20.11, 95% CI: −33.90∼−6.33, *P*=0.004). Six studies reported the mean duration of response [11, 17, 19, 22, 24, 28]. The combined statistical results showed that the duration of response in the treatment group was longer than that in the control group (*n* = 440, SMD = 3.22, 95% CI: 1.63∼4.80, *P* < 0.0001); see Figures 7 and 8.

### 3.8. Publication Bias

**Figure 9:**
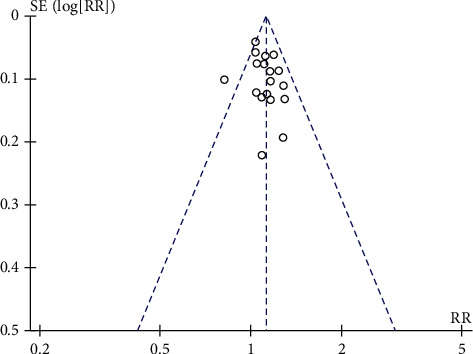
Total efficiency funnel.

Publication bias, which has always been a problem in meta-analysis, refers to the fact that research with positive results is easier to publish than research with negative results. The funnel chart analysis results of the main outcome indicators of the response rates of pain relief suggested that publication bias might exist and exaggerate the efficacy of acupuncture combined with three-step analgesic drugs in the treatment of cancer pain; see Figure 9.

## 4. Discussion

Cancer is a significant global public health issue, and the disease burden is growing. Globally, there are 18.1 million new cancer cases and 9.6 million cancer deaths each year; cancer deaths are expected to exceed 13 million by 2030, and 70% of cancer deaths globally occur in low-income and middle-income countries. In China in 2018, nearly 24% (4.3 million) of global new cases and 30% (2.9 million) of deaths occurred [29, 30]. China is the largest developing country. Chinese doctors need to pay attention to promoting cancer prevention for people and treating cancer patients. Cancer pain is severe, intolerable, and intractable pain, and such pain is a main symptom in the advanced stages of malignant tumours. When the tumour body markedly enlarges, tissue necrosis, erosion, and so on result in severe compression of, damage to, and irritation of the nerve sheath, nerve fibres, and blood vessels. Although there are many ways to treat cancer pain, many years of clinical experience at home and abroad indicate that providers believe that drug therapy is still the most common and effective way to control cancer pain. The WHO three-step cancer pain treatment programme has become an internationally accepted cancer pain drug treatment method that can control most cancer pain; however, three-step pain drugs, especially opioids, are often accompanied by side effects such as nausea, vomiting, constipation, drowsiness, dizziness, and respiratory depression [31].

There are many ways to treat cancer, but in recent years, TCM has played an increasingly important role in cancer prevention and treatment. As an integral part of TCM, acupuncture has been used to treat pain for thousands of years. The complications of acupuncture in the treatment of pain diseases are fewer than those of drug treatment [32]. Wang Limei et al. reported that [33] complications such as pneumothorax, dizziness, pain, needle syncope, infection, and visceral puncture can occur due to improper acupuncture manipulation; however, when doctors master anatomical knowledge, perform acupuncture correctly, and sterilise needles strictly, complications are further reduced. Modern research shows that the mechanism of acupuncture analgesia may be related to regulating the self-healing of the body, changing patients' perceptions of pain, and affecting the conduction of the central nervous system [34]. Another possible acupuncture mechanism is stimulation/excitation of the endogenous pain modulation system, which induces the secretion of endogenous opioids, blocks the transmission of neurotransmitters, and regulates the perception of pain to achieve analgesia [35]. A third possibility is that the pain signals from acupuncture are modulated in the pain receptor areas, and the dorsal root ganglion cells of the outgoing primary neurons transmit the signal to the near end of the secondary neurons. The pain signal produced by acupuncture may then induce the secretion of endogenous opioids and analgesia in the periaqueductal grey matter of the midbrain, or it may induce the penetration of electric ions, stimulate neurons, and exert an inhibitory effect in the intercellular area of the periaqueductal grey matter of the midbrain [36]. The exact mechanism of acupuncture's analgesic effect has not yet been elucidated. However, this review of acupuncture treatment of cancer pain with a large number of RCT experiments demonstrated that acupuncture treatment of cancer has fewer adverse reactions such as nausea and vomiting than analgesic drug treatment alone. Acupuncture treatment for cancer pain is considered to have sufficient evidence to determine its effectiveness [37]; these results are encouraging and support further research on acupuncture treatment for cancer.

## 5. Conclusion

Based on the meta-analysis of 19 studies, compared with the treatment of cancer pain with three-step analgesic drug treatment alone, the response rates of pain relief from acupuncture combined with three-step analgesic drug treatment were higher, the NRS scores were lower, the incidence of adverse reactions such as nausea and vomiting was less frequent, the incidence of times of burst pain was also less frequent, the onset time to analgesic effect was shorter, and the duration of pain response was longer.

There were several limitations in this study. The lack of high-quality studies in the literature may limit the validity of the results. Meta-analyses generally face methodological challenges such as insufficient literature retrieval, potential selection bias for which studies are included, and inappropriate evaluation of the quality of the original research. This study only included published literature and did not search for unpublished literature; in addition, there may be publication bias in the literature.

In conclusion, this study shows that acupuncture combined with three-step analgesic drugs has specific advantages over three-step analgesic drugs alone in the treatment of cancer pain. It is hoped that, in the future, rigorous randomised controlled trials will be carried out with multicentre and large-sample studies to determine acupuncture's exact curative effect and further demonstrate the superiority of acupuncture combined with three-step analgesic drugs over the use of such drugs alone to treat cancer pain.

## Figures and Tables

**Table 1:** Summary of randomised clinical studies of acupuncture combined with three-step analgesic drug therapy for the treatment of cancer pain.

Study (year)	Type of cancer	Sample sizes		Interventions		Acupuncture point selection	Session frequency and duration	Main outcomes and assessment of pain
		T	C	T	C			
Wang (2018) [10]	Various	35	35	AT + C	Drug (three-step analgesic ladder)	LI4, LR3, and Ashi point	30 min qd 6 weeks	Response rate, NRS, and side effect rate

Wang (2016) [11]	Lung cancer	30	30	EA + C	Oxycodone sustained-release tablets	LI4, PC6, ST36, and SP6	30 min qd 14 days	Response rate, NRS, side effect rate, burst pain, onset time, and DOR

Zhong (2016) [12]	Various	30	30	FA + C	Morphine sulfate sustained-release tablets	Ashi point	Once a day 14 days	Response rate, QOL, and burst pain

Mi (2010) [13]	Gastric cancer	32	30	FN + AT + C	Drug (three-step analgesic ladder)	FN: BL21, BL18, and BL17. AT: CV12, ST25, and St36	30 min qod 4 weeks	Response rate and side effect rate

Bai (2019) [14]	Various	50	50	FN + C	Drug (three-step analgesic ladder)	Ashi point, ST36, and SP6	qod 14 days	Response rate, NRS, and side effect rate

Liu (2018) [15]	Various	72	75	TEAS + C	Drug (three-step analgesic ladder)	LI4, PC6, ST36, and SP6	30 min bid 3 weeks	Response rate, BPI-S, KPS, and side effect rate

Liu (2011) [16]	Liver cancer	30	30	AT + C	Tramadol hydrochloride sustained-release tablets	SP4, PC6, GB41, TE5, SI3, BL62, LU7, KI6, LR3, and LR14	qd 14 days	Response rate, NRS, QOL, side effect rate, onset time, and DOR

Fu (2019) [17]	Various	16/16	16	AT + C	Morphine hydrochloride sustained-release tablets	PC6 and SP6	1 h qd	Response rate, NRS, KPS, side effect rate, burst pain, onset time, and DOR
				WA + C				

Wu (2019) [18]	Various	30	30	WA + C	Drug (three-step analgesic ladder)	Based on syndrome differentiation and disease differentiation	12 h qd 10 days	Response rate, VAS, burst pain, and side effect rate

Dong (2018) [19]	Various	60	60	WA + C	Drug (three-step analgesic ladder)	Based on syndrome differentiation and disease differentiation	10–12 h qd 7 days	Response rate, NRS, QLQ-C30, and side effect rate

Sun (2016) [20]	Various	30	30	AT + C	Oxycodone	LI4, PC6, ST36, SP6, Ashi point, and others	30 min qd 14 days	Response rate, NRS, KPS, QOL, and side effect rate

Zhang (2014) [21]	Various	30	30	AT + C	Drug (three-step analgesic ladder)	LI4 and ST36. Lung cancer: PC6 and LU6. Liver cancer: GB34, LR6, and LR3. Colorectal cancer: PC6, CV12, and TE6	30 min qd 7 days	Response rate, QOL, side effect rate, onset time, and DOR

Hui (2019) [22]	Various	40	40	AT + C	Drug (three-step analgesic ladder)	Ashi point, LI4, GV14, BL11, GB34, and LR3	30 min qd 14 days	Response rate, side effect rate, onset time, and DOR

Tan (2012) [23]	Various	106	101	AT + C	Drug (three-step analgesic ladder)	LI4 and PC6. Lung cancer: LU6. Liver cancer: GB34 and LR6. Colorectal cancer: CV12, ST36, and TE6	0.5–1 h qd 3 weeks	Response rate and side effect rate

Fan (2017) [24]	Lung cancer	35	34	AT + C	Drug (three-step analgesic ladder)	PC6, LI4, ST36, GB34, and SP6	20 min qd 20 days	Response rate, NRS, onset time, and DOR

Jiang (2016) [25]	Various	25	25	AT + C	Drug (three-step analgesic ladder)	Ashi point, LR3, and LI4	30 min qd 7 days	Response rate and NRS

Li (2017) [26]	Gastric cancer	30	30	AT + C	Drug (three-step analgesic ladder)	ST36, LR3, and LI4	30 min qd 7 days	Response rate, NRS, QOL, and side effect rate

Huang (2018) [27]	Various	31	31	AT + C	Drug (three-step analgesic ladder)	PC6. Lung cancer: LI4, LU4, LU6, and ST36. Liver cancer: GB34 and LR3. Breast cancer: LI4, STI8, and CV9. Gastric cancer: CV12, ST36, and TE6	30 min qd 7 days	Response rate

Peng (2012) [28]	Various	23	24	EA + C	Drug (three-step analgesic ladder)	LI4, PC6, ST36, and SP6	30 min qd 7 days	Response rate, onset time, and DOR

T: treatment group, C: control group, AT: acupuncture, EA: electroacupuncture, FA: floating acupuncture, FN: fire needle, WA: wrist-ankle acupuncture, DOR: duration of response, NRS: numerical rating scale, BPI-S: brief pain inventory-severity, QOL: quality of life, and KPS: Karnofsky performance status.

**Table 2:** Risk of bias for the 19 included studies using a modified approach to the Cochrane risk of bias tool.

	Risk of bias							Trial characteristics		
Source	Random sequence generation	Allocation concealment	Blinding of participants and personnel	Blinding of outcome assessors	Infrequent loss to follow-up	Free of selective outcome reporting	Free of other types of bias	Statistical analysis (per protocol, intention to treat, etc.)	How is loss to follow-up handled?	Adverse event
Wang (2018) [10]	Definitely yes	Definitely yes	Probably no	Probably no	Definitely no	Definitely no	Definitely no	Not mentioned	Ignored	Yes

Wang (2016) [11]	Probably yes	Probably yes	Probably no	Probably no	Definitely yes	Definitely yes	Probably yes	Not mentioned	Not mentioned	Yes

Zhong (2016) [12]	Definitely yes	Definitely yes	Probably no	Probably no	Definitely yes	Definitely no	Probably yes	Per protocol	Not mentioned	No

Mi (2010) [13]	Definitely yes	Definitely yes	Probably no	Probably no	Definitely yes	Definitely yes	Probably yes	Not mentioned	Not mentioned	Yes

Bai (2019) [14]	Definitely yes	Definitely yes	Probably no	Probably no	Definitely yes	Definitely yes	Probably yes	Not mentioned	Not mentioned	Yes

Liu (2018) [15]	Definitely yes	Definitely yes	Probably no	Probably no	Definitely no	Definitely yes	Probably yes	Not mentioned	Ignored	Yes

Liu (2011) [16]	Definitely yes	Definitely yes	Probably no	Probably no	Definitely yes	Definitely yes	Probably yes	Not mentioned	Not mentioned	Yes

Fu (2019) [17]	Definitely yes	Definitely yes	Probably no	Probably no	Definitely no	Definitely no	Definitely no	Per protocol	Ignored	Yes

Wu (2019) [18]	Definitely yes	Definitely yes	Probably no	Probably no	Definitely yes	Definitely no	Probably yes	Not mentioned	Not mentioned	Yes

Dong (2018) [19]	Probably yes	Probably yes	Probably no	Probably no	Definitely yes	Definitely yes	Probably yes	Not mentioned	Not mentioned	Yes

Sun (2016) [20]	Probably yes	Probably yes	Probably no	Probably no	Definitely yes	Definitely yes	Probably yes	Not mentioned	Not mentioned	Yes

Zhang (2014) [21]	Probably yes	Probably yes	Probably no	Probably no	Definitely yes	Definitely yes	Probably yes	Per protocol	Not mentioned	Yes

Hui (2019) [22]	Definitely yes	Definitely yes	Probably no	Probably no	Definitely yes	Definitely yes	Probably yes	Not mentioned	Not mentioned	Yes

Tan (2012) [23]	Probably yes	Probably yes	Probably no	Probably no	Definitely yes	Definitely no	Probably yes	Not mentioned	Not mentioned	Yes

Fan (2017) [24]	Definitely yes	Definitely yes	Probably no	Probably no	Definitely yes	Definitely yes	Probably yes	Per protocol	Not mentioned	No

Jiang (2016) [25]	Probably yes	Probably yes	Probably no	Probably no	Definitely yes	Definitely no	Probably yes	Per protocol	Not mentioned	No

Li (2017) [26]	Probably yes	Probably yes	Probably no	Probably no	Definitely yes	Definitely yes	Probably yes	Not mentioned	Not mentioned	Yes

Huang (2018) [27]	Probably yes	Probably yes	Probably no	Probably no	Definitely yes	Definitely yes	Probably yes	Not mentioned	Not mentioned	No

Peng (2012) [28]	Probably yes	Probably yes	Probably no	Probably no	Definitely yes	Definitely yes	Probably yes	Per protocol	Not mentioned	No

## Data Availability

The data can be obtained from the author upon reasonable request.
